# Analysis of QTLs and Candidate Genes for Tassel Symptoms in Maize Infected with *Sporisorium reilianum*

**DOI:** 10.3390/ijms232214416

**Published:** 2022-11-20

**Authors:** Yu Zhou, Minhao Yao, Qian Wang, Xiaoming Zhang, Hong Di, Lin Zhang, Ling Dong, Qingyu Xu, Xianjun Liu, Xing Zeng, Zhenhua Wang

**Affiliations:** Key Laboratory of Germplasm Enhancement, Physiology and Ecology of Food Crops in Cold Region, Engineering Technology Research Center of Maize Germplasm Resources Innovation on Cold Land of Heilongjiang Province, Northeast Agricultural University, Harbin 150030, China

**Keywords:** maize, *Sporisorium reilianum*, tassel symptoms, bulked segregation analysis, QTL

## Abstract

Heat smut is a fungal soil-borne disease caused by *Sporisorium reilianum*, and affects the development of male and female tassels. Our previous research found that the tassel symptoms in maize infected with *Sporisorium reilianum* significantly differed in inbred lines with Sipingtou blood, and exhibited stable heredity over time at multiple locations. In this study, cytological analysis demonstrated that the cellular organization structures of three typical inbred lines (Huangzao4, Jing7, and Chang7-2) showed significant discrepancies at the VT stage. QTLs that control the different symptoms of maize tassels infected with *Sporisorium reilianum* were located in two F_2_ populations, which were constructed using three typical inbred lines. The BSA (bulked segregation analysis) method was used to construct mixed gene pools based on typical tassel symptoms. The QTLs of different symptoms of maize tassels infected with *Sporisorium reilianum* were detected with 869 SSR markers covering the whole maize genome. The mixed gene pools were screened with polymorphic markers between the parents. Additional SSR markers were added near the above marker to detect genotypes in partially single plants in F_2_ populations. The QTL controlling tassel symptoms in the Huangzao4 and Jing7 lines was located on the bin 1.06 region, between the markers of umc1590 and bnlg1598, and explained 21.12% of the phenotypic variation with an additive effect of 0.6524. The QTL controlling the tassel symptoms of the Jing7 and Chang7-2 lines was located on the bin 2.07 region, between the markers of umc1042 and bnlg1335, and explained 11.26% phenotypic variation with an additive effect of 0.4355. Two candidate genes (*ZmABP2* and *Zm00001D006403*) were identified by a conjoint analysis of label-free quantification proteome sequencings.

## 1. Introduction

Maize (*Zea mays* L.) is the most widely planted crop in the world. However, yield loss in maize typically occurs because of head smut, a systemic fungal disease caused by *Sporisorium reilianum* [1,2]. Field management or bactericidal seed coating can be used to control head smut, but these strategies are costly, unstable, and pollute the environment. Therefore, exploring the molecular mechanisms of smut resistance and identifying new smut-resistant varieties is another method of preventing head smut. Once infected with head smut, it can be difficult or impossible to harvest maize plants. Breeding smut-resistant hybrids is the safest and most economical way of reducing yield loss [3].

The inheritance of resistance to maize head smut is a quantitative trait controlled by multiple genes and is stably inherited [4,5]. Since 1999, researchers have performed experiments on the mapping and functional analysis of genes that resist heat smut in maize, using recombinant inbred lines, F_2_ isolates, or backcrossed populations. The major QTL for resisting head smut is located on bin 2.09, with a BC_1_ population constructed by Ji1037×Huangzao4, and explains 36% of phenotypic variation [6,7]. Furthermore, combining the results of fine localization of recombinant individuals and inbred line association analysis, the major *qSH2.09* on chromosome 2 was located between the SNP molecular markers, PZE-102187486 and PZE-102188421, with a physical distance of approximately 1.0 Mb [8]. Finally, using the backcrossed population constructed by Ji1307 and Huangzao4, the primary effect of QTL-*QHSR1* was precisely mapped, and five disease resistance-related candidate genes were predicted. The major gene for head smut resistance, *ZmWAK*, was found on bin 2.09 [9], whereas the NBS-LRR resistance gene *ZmNL*, which is related to smut resistance, was also identified in this region [10].

Pathogenic bacteria can cause specific diseases in plants, often due to complex interactions between plants and pathogenic bacteria that alter the original cell morphology, growth, and metabolism of the host plant [11]. Plants resist the invasion of pathogens through innate or acquired immune systems to survive under biological stress [12]. The host’s original metabolic process changes the tissue and cell morphology, causing disease-specific symptoms [13]. At the seedling stage, maize is infected with *Sporisorium reilianum*, while the spores of pathogenic bacteria are first produced in inflorescences [14]. Molecular mechanisms of resistance and pathogenesis of plant disease are very complex interaction systems [15]. The primary plant defense and tissue of plants will be interfered with and destroyed by pathogens [16]. Studying the symptomatic mechanisms can help to reveal the mechanism behind disease formation, analyze the interaction between plants and pathogens, and prevent disease occurrence [17,18]. The main symptoms of head smut include deformation and swelling of the ear with *Sporisorium reilianum* and different symptoms of tassel [19,20,21]. The symptoms of head smut cannot be observed until the tassels and ears emerge [22].

In this study, cytological analysis was performed to identify changes in the cellular organization structures of three typical inbred lines (Huangzao4, Jing7, and Chang7-2) from the VE to VT stage. To identify the QTLs that control tassel symptoms of maize infecting *Sporisorium reilianum*, two F_2_ populations were constructed with Huangzao4 and Jing7, and Jing7 and Chang7-2. The candidate genes were identified through QTL mapping and label-free quantification proteome sequencing.

## 2. Results

### 2.1. Sporisorium reilianum Causes Damage to Maize Cell Structure

The inbred lines with Sipingtou blood were classified into three types, according to the tassel symptoms during maturation, and were artificially inoculated with *Sporisorium reilianum*. Class A entails several tassel carbonizations, with black filaments and a large number of spores, with typical symptoms of Huangzao4; Class B is the overall expansion of the tassel without spores, with typical symptoms of Jing7; and Class C is when the base of the tassel is afflicted while the upper part develops typical Chang7-2 symptoms.

**Figure 1:**
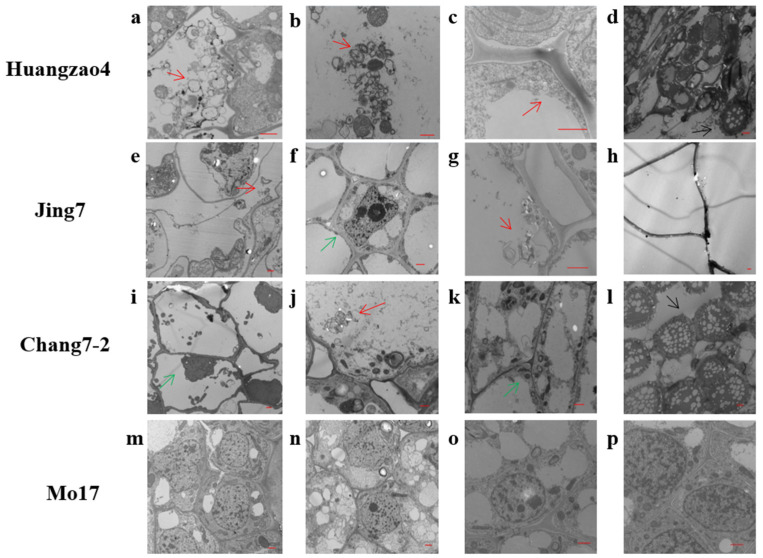
Cell structure analysis of three typical maize inbred lines and the resistant inbred line Mo17 at different growth stages. (**a**–**d**) Cells from Huangzao4 growth points or tassels after inoculation at the VE, V8, V12, and VT stages, respectively. (**e**–**h**) Cells from Jing7 growth points or tassels after inoculation at the VE, V8, V12, and VT stages, respectively. (**i**–**l**) Cells from Chang7-2 growth points or tassels after inoculation at the VE, V8, V12, and VT stages, respectively. (**m**–**p**) Cells from Mo17 growth points or tassels after inoculation at the VE, V8, V12, and VT stages, respectively. The ”-”scale is 1 μm. The red arrow indicates cell autophagy; the green arrow indicates plasmolysis.

The cell structure of maize inbred lines infected with *Sporisorium reilianum* (Huangzao4, Jing7, Chang7-2, and resistant inbred line Mo17) were analyzed (Figure 1a–l). The resistant inbred line Mo17 showed normal cell structure at each growth stage (Figure 1m–p). However, cell morphology related to the three typical symptoms was consistent in the three typical inbred lines from the VE to V8 stage (Figure 1a,b,e,f,i,j). In the V12 stage, Huangzao4 and Chang7-2 showed slight cell wall degradation (Figure 1c,k), whereas no significant changes were observed in Jing7 (Figure 1g). In the VT stage, Huangzao4 and Chang 7-2 had similar cell morphology at the susceptible site, with many *Sporisorium reilianum* spores, and their cell structures were destroyed (Figure 1d,l). The contents of Jing7 cells were completely lost, with only the cell wall remaining, but few *Sporisorium reilianum* spores were found (Figure 1h).

For Class A Huangzao4 cells infected with *Sporisorium reilianum*, plasmolysis and autophagy were observed from the VE to V8 stage, during which they retained an intact cell structure. The cell walls of Huangzao4 disintegrated at the V12 stage. At the VT stage, the cells of Huangzao4 developed severe lesions inside, with *Sporisorium reilianum* spores forming in large numbers and destroying cell walls, leaving only fibrous material (Figure 1a–d). After the inoculation of Jing7, plasmolysis and autophagy were also observed from the VE to the V12 stage, but overall, cells maintained an intact cell structure. At the VT stage, the cell contents of Jing7 were completely lost, with only the cell wall remaining, and a few *Sporisorium reilianum* spores were found (Figure 1e–h). After the inoculation of Chang7-2, plasmolysis and autophagy occurred from the VE to V8 stage at growth points or tassel cells. Cell wall disintegration occurred at the V12 stage, and many *Sporisorium reilianum* spores appeared at the susceptible site at the VT stage, during which the cell wall was disrupted (Figure 1i–l). However, for the resistant inbred line Mo17, the cell structure was normal at each growth stage (Figure 1m–p).

The permeability of the host cell membrane experienced plasmolysis due to *Sporisorium reilianum* infections. Typical head smut symptoms could be related to cell wall degradation, and formation of the *Sporisorium reilianum* spore, which is the primary reason for changes in the phenotype of maize infected with *Sporisorium reilianum*.

### 2.2. Identification of Tassel Symptoms in the Two F_2_ Populations

**Figure 2:**
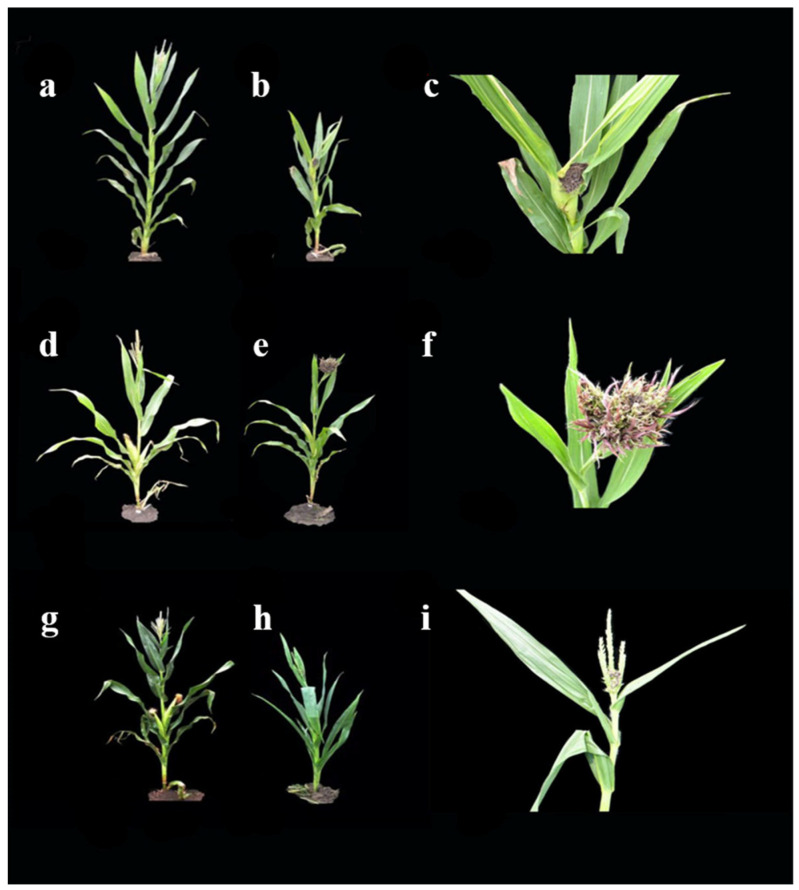
Three types of tassel symptoms in typical maize inbred lines infected with *Sporisorium reilianum*: (**a**) Huangzao4 normal plant without *Sporisorium reilianum*; (**b**) Huangzao4 plant infected with *Sporisorium reilianum*; (**c**) Class A with typical symptoms of the tassel of Huangzao4; (**d**) Jing7 normal plant without *Sporisorium reilianum*; (**e**) Jing7 plant infected with *Sporisorium reilianum*; (**f**) Class B with typical symptoms of the tassel of Jing7; (**g**) Chang7-2 normal plant without *Sporisorium reilianum*; (**h**) Chang7-2 plant infected with *Sporisorium reilianum*; and (**i**) Class C with typical symptoms of the tassel of Chang7-2.

**Figure 3:**
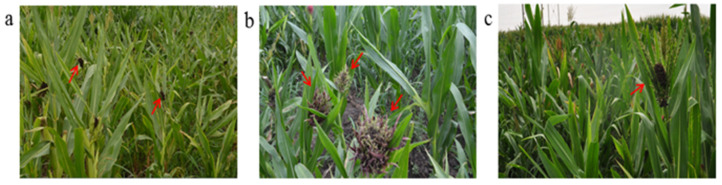
Different tassel symptoms of plants infected with *Sporisorium reilianum* in F_2_ populations: (**a**) typical Huangzao4 plants in Huangzao4×Jing7 F_2_ population; (**b**) typical Jing7 plants in the Huangzao4×Jing7 F_2_ population; (**c**) typical Chang7-2 plants in the Jing7×Chang7-2 F_2_ population.

Using the three typical maize lines, two F_2_ populations (Huangzao4×Jing7 and Jing7×Chang7-2) were constructed based on three maize inbred lines, Huangzao4 (Figure 2a–c), Jing7 (Figure 2d–f), and Chang7-2 (Figure 2g–i). These plants showed different symptoms in the tassel of maize infected with *Sporisorium reilianum*. Phenotypic identification of F_2_ plants was performed according to the symptoms of tassels infected with *Sporisorium reilianum*. The statistical results are shown in Table 1. In the Huangzao4×Jing7 F_2_ population, there were 265 plants with Class A tassel symptoms, 234 plants with Class B tassel symptoms, and 890 plants with Class D tassel symptoms (Supplementary Figure S1a,b). In the Jing7×Chang7-2 F_2_ population, there were 146 plants with Class B tassel symptoms, 183 plants with Class C tassel symptoms, and 263 plants with Class E tassel symptoms (Supplementary Figure S1c). According to the chi-square test, the number of the parent phenotype and intermediate type did not follow a 1:2:1 ratio in each population. There were several intermediate individual plants in each F_2_ population, and it was difficult to divide them and classify the occurrence of the leafy tufted half spike (Figure 3a–c). These results demonstrated that these traits were not discrete gene characteristics, making the intermediate phenotype more difficult to distinguish.

### 2.3. Identification of Polymorphisms between the Parents

**Figure 4:**
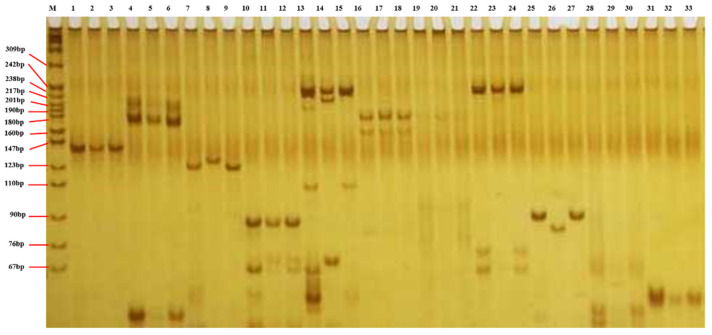
SSR marker screening among parents. Electrophoresis lanes: M is Marker pBR322 DNA/MspⅠ; the samples are from Huangzao4, Jing7, and Chang7-2, respectively; 1, 2, and 3 are bnlg1083; 4, 5, and 6 are bnlg1614; 7, 8, and 9 are bnlg1953; 10, 11, and 12 are umc1169; 13, 14, and 15 are bnlg1458; 16, 17, and 18 are umc1321; 19, 20, and 21 are umc1073; 22, 23, and 24 are umc1114; 25, 26, and 27 are phi078; 28, 29, and 30 are umc1944; 31, 32, and 33 are umc1095.

Polymorphism analysis was performed in three parental inbred lines using 869 pairs of SSR markers covering the whole maize genome in the IBM2 2008 Neighbors genetic linkage map (http://www.miazegdb.org, accessed on 28 October 2017) (Figure 4). The results showed that there were 207 pairs of polymorphic markers between Huangzao4 and Jing7, with a polymorphic rate of 23.8%. There were 162 pairs of polymorphic markers between Huangzao4 and Chang7-2, with a polymorphic rate of 18.6%; there were 235 pairs of polymorphic markers between Jing7 and Chang7-2, with a polymorphic rate of 27.0%. Based on these results, two F_2_ populations, Huangzao4×Jing7 and Jing7×Chang7-2, were constructed using two inbred lines with higher polymorphisms to localize the QTLs of maize infected with *Sporisorium reilianum*. The best stable SSR markers were used to screen the polymorphisms of mixed gene pools.

### 2.4. Construction of Mixed Gene Pools and Selection of Primers

**Figure 5:**
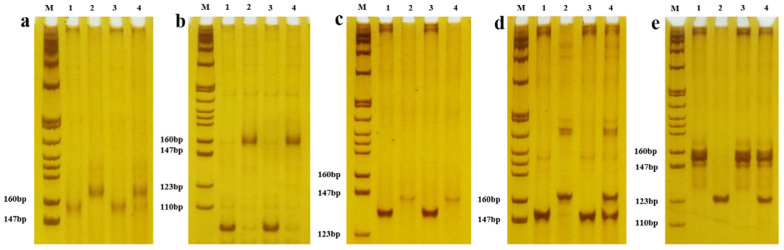
Screening of SSR markers between extreme phenotype DNA pools. Electrophoresis lanes: M is Marker pBR322 DNA/MspI; (**a**) molecular marker of umc1601 and 1, 2, 3, and 4 are Huangzao4, Jing7, Huangzao4 types of the mixed gene pool, and Jing7 types of the mixed gene pool, respectively; (**b**) molecular marker of umc1590 and 1, 2, 3, and 4 are Huangzao4, Jing7, Huangzao4 types of the mixed gene pool, and Jing7 types of the mixed gene pool; (**c**) molecular marker of umc1754 and 1, 2, 3, and 4 are Huangzao4, Jing7, Huangzao4 types of the mixed gene pool, and Jing7 types of the mixed gene pool; (**d**) molecular markers of bnlg1914 and 1, 2, 3, and 4 are Jing7, Chang7-2, Jing7 types of the mixed gene pool, and Chang7-2 types of the mixed gene pool; and (**e**) molecular marker of bnlg1335 and 1, 2, 3, 4 are Jing7, Chang7-2, Jing7 types of the mixed gene pool, and Chang7-2 types of the mixed gene pool.

For the F_2_ populations of Huangzao4×Jing7, the mixed gene pools AB-A and AB-B were built with 15 lines of extreme A phenotypes and 15 lines of extreme B phenotypes, respectively. Three pairs of polymorphism primers, umc1601 (bin1.05), umc1590 (bin1.06), and umc1754 (bin1.06), were polymorphic between Huangzao4 and Jing7 males in mixture pools of extreme tassel symptoms (Figure 5a–c). For the F_2_ populations of Jing7×Chang7-2, the mixed gene pools BC-B and BC-C were built with 15 lines of extreme B phenotypes and 15 lines of extreme C phenotypes, respectively. Two pairs of polymorphism primers, bnlg1914 (bin2.05) and bnlg1335 (bin2.07), were polymorphic between Jing7 and Chang7-2 male mixture pools of extreme tassel symptoms (Figure 5d,e).

To further analyze this relationship, we combined the above five primer pairs and controlled for the tassel symptoms related to *Sporisorium reilianum* infections to identify 20 polymorphic SSR markers from the database of both Maize Genetics and Genomics (http://www.maizegdb.org/, accessed on 10 January 2018) in the regions of umc1601, umc1590, umc1754, bnlg1914, and bnlg1335. The polymorphism analysis from the parents showed that five SSR markers (umc1395, umc1811, umc2057, bnlg1598, and umc1323) from chromosome 1 were polymorphic between Huangzao4 and Jing7. Three SSR markers (umc2019, umc2205, and umc1042) from chromosome 2 were polymorphic between Jing7 and Chang7-2. The distribution of the above primers on the chromosome, according to the IBM2 2008 Neighbors map and the B73 sequencing results, is shown in Supplementary Table S1.

### 2.5. Preliminary Mapping of QTLs

**Figure 6:**
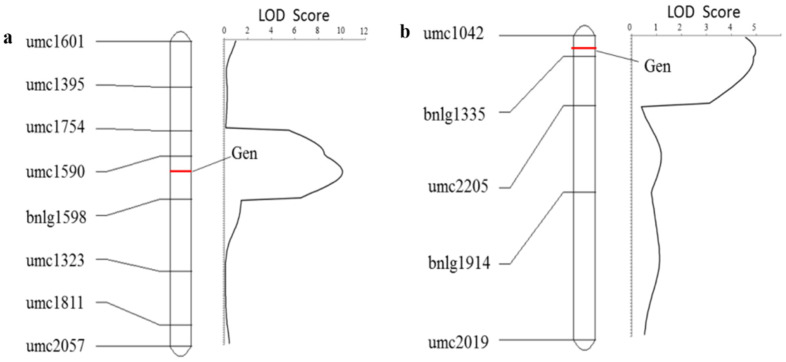
QTL results of tassel symptoms of maize infected with *Sporisorium reilianum* in two F_2_ populations. (**a**) QTL of tassel symptoms of maize infected with *Sporisorium reilianum* between Huangzao4 and Jing7. (**b**) QTL of tassel symptoms of maize infected with *Sporisorium reilianum* between Jing7 and Chang7-2.

In the F_2_ population of Huangzao4×Jing7, 270 plants with identified phenotypes were used for QTL mapping with eight SSR polymorphic markers (umc1395, unc1601, umc1323, umc1754, umc1590, umc1811, umc2057, and bnlg1598), using the IciMapping4.0 mapping software (Figure 6a). The total length of the map was 125.67 cM, and the average distance between markers was 17.95 cM. The minimum distance was 8.78 cM, between the markers umc1811 and umc2057, and the maximum distance was 29.77 cM, between the markers bnlg1598 and umc1323. In the bin 1.06 region on chromosome 1, there was a major QTL controlling the symptoms of tassels infected by *Sporisorium reilianum* in umc1590 and bnlg1598, and the genetic map distance was 6.67 and 11.1 cM, respectively, with a LOD value of 10.0437. This explained 21.12% of the phenotypic variation rate, with an additive effect of 0.6524. The isolates of the umc1590 and bnlg1598 genotypes we obtained were detected by a chi-square test in the Huangzao4×Jing7 F_2_ population using reliable phenotypic values. The results demonstrated that the umc1590 genotype was biased toward the Huangzao4 genotype, whereas the bnlg1598 genotype did not deviate.

In the F_2_ population of Jing7×Chang7-2, 202 plants with identified phenotypes were used for QTL mapping with five SSR polymorphic markers (umc1914, umc2019, umc2205, umc1335, and umc1042), using the IciMapping4.0 mapping software (Figure 6b). The total length of the F_2_ population of Jing7×Chang7-2 was 90.69 cM, with an average distance between markers of 22.67 cM. The minimum distance was 6.1 cM, between the markers umc1042 and umc1335, and the maximum distance was 43.91 cM, between the markers bnlg1914 and umc2019. In the bin 2.07 region on chromosome 2, there was a major QTL controlling the symptoms of tassels infected by *Sporisorium reilianum* from umc1042 to bnlg1335, and the genetic map distance was 4 and 2.1 cM, with a LOD value of 4.9755. This can explain 11.26% of the phenotypic variation rate, with an additive effect of 0.4355. The isolates of the umc1042 and bnlg1335 genotypes were detected by a chi-square test in the Jing7×Chang7-2 F_2_ population with reliable phenotypic values. The results demonstrated that the umc1042 and bnlg1335 genotype was biased toward the Jing7 genotype.

### 2.6. Predicting Candidate Gene Related to Tassel Symptoms of Maize Infected with Sporisorium reilianum

In the interval from umc1590 to bnlg1598 on bin 1.06, there were no annotated genes. However, there were 72 genes detected from umc1042 to bnlg1335 on the bin 2.07 region. Two candidate genes from umc1042 to bnlg1335, *ZmABP2* and *Zm00001d006403*, were found to be directly associated with tassel symptoms in maize infected with *Sporisorium reilianum*, combined with differentially expressed protein analysis between Jing7 and Chang7-2 of label-free quantification proteome sequencing.

**Figure 7:**
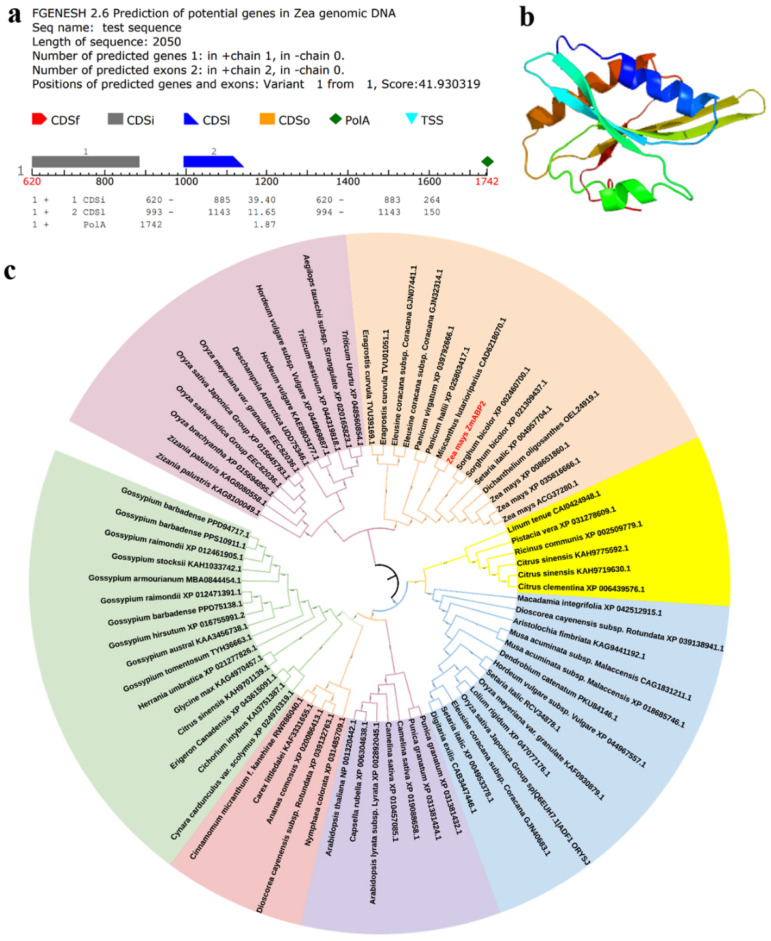
Bioinformatics analysis of the candidate gene *ZmABP2*. (**a**) Gene prediction of *ZmABP2* gene (TSS: Transcription start site; CDSf: Start exon; CDSI: Terminal exon). (**b**) Tertiary structure of ZmABP2 protein. (**c**) Phylogenetic analysis of ZmABP2 protein.

The functional annotation of the *ZmABP2* gene was actin depolymerizing factor 2. The *ZmABP2* gene was located on the negative chain, with a total length of 1050 bp, including two exons. The transcription started at 620 bp and ended at 1742 bp. It had a typical tail signal and was a complete gene (Figure 7a). The tissue with the highest expression of the *ZmABP2* gene was mature pollen (FPKM = 1946.7) (Supplementary Figure S2a). The structure of the ZmABP2 protein was predicted and analyzed. Its secondary structure was mainly composed of α-helix (34%), followed by β-chain (27%), and irregular crimp (15%) structures. The encoded protein of ZmABP2 is an acidic protein including 140 amino acids, with a relative molecular weight of 16,211.22 kD and an isoelectric point of 5.57 (Supplementary Figure S2b and Figure 7b). The conservative domain of the ZmABP2 protein was predicted using the InterPro online website. The ZmABP2 protein belongs to the ADF-H superfamily, and the actin depolymerization factor homology (ADF-H) domain is a ~150 amino acid motif, which exists in three types of eukaryotic actin-binding proteins with different phylogenetic development. GO analysis found that its functions in biological processes were actin filament depolymerization (GO: 00300422), molecular functional actin binding (GO: 0003779), and cell component actin cytoskeleton (GO: 0015629) (Supplementary Figure S2c). The ZmABP2 protein did not exist in a transmembrane domain and belonged to non-transmembrane globular proteins. The signal peptide sequence was found in the ZmABP2 protein (Sec/SPI = 0.003), indicating that the protein might not be a typical secreted protein. Homologous evolution analysis showed that the *ZmABP2* gene in maize showed the highest homology, with LOC8080684 of sorghum, with a sequence similarity of 97.14% (Figure 7c).

**Figure 8:**
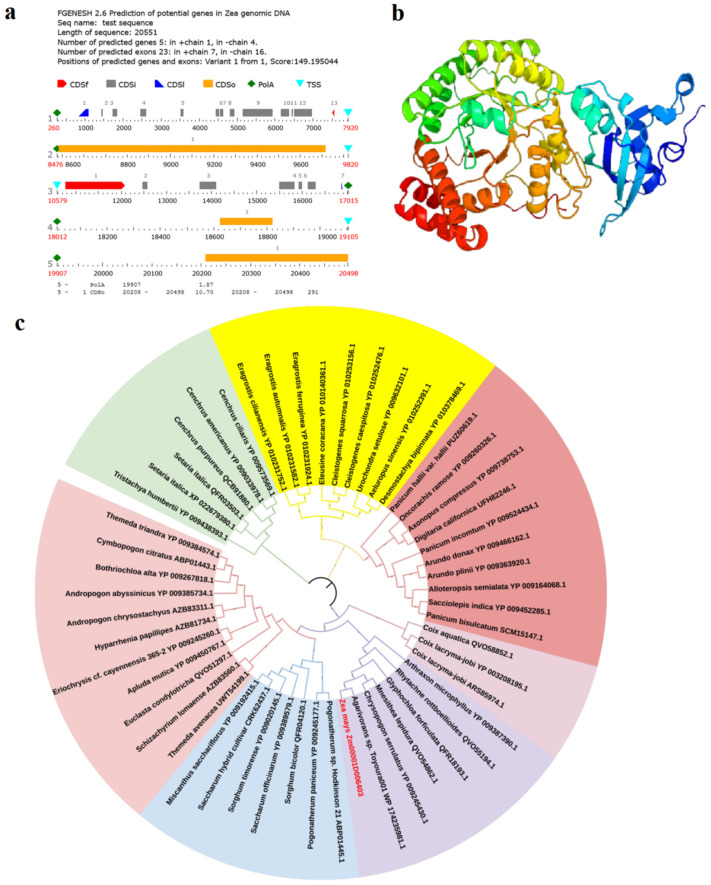
Bioinformatics analysis of the candidate gene *Zm00001d006403*. (**a**) Gene prediction of *Zm00001d006403* gene (TSS: Transcription start site; CDSf: Start exon; CDSI: Terminal exon). (**b**) Tertiary structure of Zm00001d006403 protein. (**c**) Phylogenetic analysis of Zm00001d006403 protein.

The functional annotation of *Zm00001d006403* was ATP synthase subunit beta, chloroplastic. The *Zm00001d006403* gene was located on the negative chain, with a total length of 19,551 bp, including 23 exons. The transcription started at 260 bp and ended at 20,498 bp. It had a typical tail signal and was a complete gene (Figure 8a). The *Zm00001d006403* gene was only expressed on mature leaves and silk (FPKM = 4.9 and 1.2) (MaizeGDB, https://www.maizegdb.org, accessed on 12 May 2022) (Supplementary Figure S3a). The secondary structure of the encoded protein was mainly composed of α-helix (43.0%), irregular curl (9%), and β-chain (14%) structures. The encoded protein of Zm00001d006403 was a basic protein with 483 amino acids, with a relative molecular weight of 53,477.96 kD and an isoelectric point of 6.33. The encoded protein was a basic protein with unstable hydrophobic ability (Supplementary Figure S3b and Figure 8b). The encoded protein of Zm00001d006403 belongs to an overlapping homologous superfamily, ribose diphosphate carboxylase, a large subunit, and the C-terminal domain superfamily. Ribose diphosphate carboxylation (Rubisco) plays an important role in the Calvin-reducing pentose phosphate pathway; it catalyzes the main CO2 fixation step. GO analysis found that its function in biological processes was carbon fixation (GO: 0015977), and its molecular function related to ribose bisphosphate carboxylase activity (GO: 0016984) and magnesium ion binding (GO: 0000287) (Supplementary Figure S2c). The protein did not exist in the transmembrane domain and belonged to a non-transmembrane globular protein. No signal peptide sequence was found in this protein (Sec/SPI = 0.003), indicating that the protein might not be a typical secreted protein. Homologous evolution analysis showed that the *Zm00001d006403* gene in maize showed the highest homology with NC_ 036702 of indica, with a sequence similarity of 96.85% (Figure 8c).

## 3. Discussion

### 3.1. Differences in Tassel Symptoms Infected by Head Smut of Sipingtou Bloodlines of Maize

Previous studies have found that in maize, *Sporisorium reilianum* primarily affects the flower organ, where the ear swells, shortens, and develops smut spores in the bracts [23]. In severe cases, the tassel forms either galls and black powder spores or a leaf-like cluster that does not produce black powder spores [9,24]. The results of 304 maize inbred lines inoculated with *Sporisorium reilianum* for 4–6 years have shown that some high resistance lines were found to have an average incidence of less than 2.5%, including Mo17, Liao1311, and B70. Resistance lines, such as 330, J63, and 7091 were found to have an average incidence of 8.58 to 11.56% [25], with genetic stability and little difference across the years. Inbred resistance lines of head smut Mo17 have been reported to have incidences lower than 5%. Meanwhile, the incidence of the sensitive inbred line Huangzao4 ranged from 55 to 89% in dozens of trials conducted over five years. These results demonstrate that disease incidence among inbred lines was stable, and was slightly affected by environmental factors, such as the year and region. In this study, we found that the symptoms in the ear stabilized after inoculation with *Sporisorium reilianum*. The tassel symptoms were classified into three types: Class A entails several tassel carbonization with black filaments and a large number of spores, with typical symptoms of Huangzao4; Class B is the overall expansion of the tassel without spores, with the typical symptoms of Jing7; and Class C is when the base of the tassel is afflicted while the upper part develops normally. This demonstrates that the type of head smut can be stably expressed and is related to genetic factors.

When plants are infected with pathogens, various stable phenotypes can be produced through different genetic materials [26]. The occurrence of the disease is closely related to plant genetics, such as asymptomatic, necrotic, and mosaic diseases that are caused by SMV infection of soybeans, which could be controlled by multiple alleles [27]. However, there is no physiological specialization of *Sporisorium reilianum* [28], and sequence analysis of the pathogen indicates that there is no significant difference between pathogens causing different diseases in maize [29]. This ensures that the tassels of inbred Sipingtou lines were stable after being inoculated with *Sporisorium reilianum*. Under different environmental conditions, maize inbred lines can interfere with the development of tassel symptoms due to varying severity of the disease, including incomplete panicle development. However, most Sipingtou inbred lines are highly susceptible to inbred lines, meaning the disease symptoms were stable. Although the incidence rates of head smut of Sipingtou inbred lines were indifferent, the lines could be classified by tassel symptoms in maize infected with *Sporisorium reilianum*.

### 3.2. Advantage of Using the BSA Method to Analyze Tassel Symptoms in Maize Infected with Sporisorium reilianum

The BSA method can quickly locate QTLs and target trait genes and has been widely used for gene mapping, including genome-wide association studies by batch segregation analysis of parental populations, mutant gene sequencing, and extreme variant-based aggregation. The BSA method was first used to save time when creating near-isogenic lines (NILs). This method was widely used in selfing and cross-pollinating crops to overcome various limitations when creating corresponding NILs [30,31,32]. The BSA method was also widely used for maize resistance to gray spots and stem rot [33]. In its early stages, it was mainly used to map genes with a relatively large impact on agronomically important traits, such as grain yield [34], drought tolerance [35], and water-stress tolerance in wheat [36]. As next-generation sequencing technologies improve, BSA became a popular method of quickly detecting associated markers and candidate genes by sequencing parents and offspring with extreme phenotypes. QTL-seq has been successfully used to identify early-flowering QTLs in cucumbers [37]. DNA-seq BSA has also been used to analyze cotton mutants (such as multiplex-branch and short-fiber mutants) [38], genetically map grain protein content [39], and assess yellow rust resistance in wheat [40]. BSA can help improve plant breeding by developing markers, analyzing agronomic genomics, assisting marker selection, and identifying selective phenotypes.

In this study, the BSA method was used to construct mixed pools based on the typical symptoms affecting the tassels of maize infected with *Sporisorium reilianum*, which were continuously distributed in the two F_2_ populations. A total of 15 lines with extreme phenotypes from the F_2_ population were selected and placed into mixed gene pools to screen for polymorphisms and reduce false positives. The genotypic identification of more than 200 F_2_ progenies from each population was sufficient to complete primary positioning [41]. Based on the advantage of the BSA method and the particularity of phenotype of tassel symptoms of head smut, two QTLs related to tassel symptoms of maize infected with *Sporisorium reilianum* were located.

### 3.3. Genetic Study of Maize Tassels Infected with Sporisorium reilianum

Head smut-resistant QTLs were identified from all the 10 chromosomes in maize, with a major resistance QTL on bin 2.09 [6,7,8,42,43,44]. Other minor QTL effects were reported on bins 1.01–1.06, 1.09, 2.04–2.05, 2.07–2.10, 3.04–3.05, 4.01, 4.03, 4.08, 5.03, 5.05, 6.06, 6.07, 7.01, 8.02–8.03, 8.05, 9.03, and 10.03–10.05 [6,42,43,45]. However, there were few consistent QTLs, except for a major resistance QTL on bin 2.09. Several kinds of molecular markers, such as SSRs, dCAPs, and SNPs, were identified and used to analyze head smut-resistant lines (Mo17, Qi319, and Ji1037) and smut-susceptible (Huangzao4 and Ji154, 444) lines [7,8,46,47]. *ZmWAK* is the major resistance gene on bin 2.09 and is cloned from the Mo17 line and highly expressed on mesocotyl, encoding a cell wall-related receptor kinase that can induce disease responses [48]. Based on the constructed inoculation and identification of the Huangzao4×Jing7 F_2_ and Jing7×Chang7-2 F_2_ populations, the maturity of black-spotted fungus is hereditary, there are many intermediate-type individuals in their offspring, and the symptoms afflicting their tassels are difficult to distinguish between parents. The number of different dominant types was compared using the chi-square test, which found that the seed-parent and intermediate types did not follow a 1:2:1 separation ratio, and the traits were considered to be QTLs. The BSA method was used to detect QTLs from the F_2_ population with distinct phenotypes related to the tassels of maize infected with *Sporisorium reilianum*, using 869 pairs of SSR markers covering the whole maize genome. Two QTLs controlling different tassel symptoms due to *Sporisorium reilianum* infections were located between umc1590 and bnlg1598 on bin 1.06, and umc1042 and bnlg1335 on bin 2.07. The interval from umc1590 to bnlg1598 of bin 1.06 overlapped with the reported locus from csu61 to csu92 [43]. The interval from umc1042 to bnlg1335 of bin 2.07 included the reported molecular marker bnlg1045 [49]. In summary, the two located QTLs in this study did not only connect to resistance of heat smut, but was also related to the tassel symptoms and dysorganoplasia in maize infected with *Sporisorium reilianum.* The two candidate genes related to the tassel symptoms of head smut in this study were all close to the molecular marker umc1042.

### 3.4. Function of Two Candidate Genes Related to the Tassel Symptoms of Head Smut

The candidate gene *ZmABP2* belongs to the ADF family, and the ADF proteome is thought to control actin polymerization and depolymerization in response to intracellular and extracellular signals. The plant ADF family plays a key role in growth, development, and defense-related functions, including pollen tube development, cotton fiber development, root, pathogen defense, and drought tolerance [50]. The ADF/cofilins could alter multicellular development by controlling activity in the nucleus or cytoplasm of gene expression [51]. The ZmABP protein can bind monomeric actin (g-actin) and filamentous actin (f-actin). Reorganization of the actin cytoskeleton occurs during the dormancy of pollen grains and activation of pollen tube growth, which affects the pollen development of plants [52]. In this research, the candidate gene *ZmABP2* was identified via QTL mapping and proteome sequencing. Inflorescence and branching architectures of maize can be changed when infected with *Sporisorium reilianum* [19]. Considering the function of the *ZmABP2* gene, it may be a candidate gene for tassel symptoms in maize infected with *Sporisorium reilianum*, which refers to the meristem determinacy in tassel.

Another candidate gene, *Zm00001D006403*, is a ribose-1,5-bisphosphate carboxylase/oxygenase (Rubisco) large subunit-related gene. Rubisco catalyzes the rate-limiting step of CO2 fixation in photosynthesis and is therefore a key enzyme in the global carbon cycle [53,54,55]. Rubisco is highly regulated by environmental fluctuations in response to abiotic stresses [56]. Studies have demonstrated that increasing the content of Rubisco-active enzymes can improve the yield and persistence of rice at high temperatures without reducing Rubisco content [57]. Many experiments have demonstrated that the promoter of the rice rbcS (small subunit of RuBisCO) gene contributes to the expression of target genes limited to green tissues, and that the marker-free S6 RNAi transgenic lines driven by the green tissue expression promoter of rice rbcS have great potential for breeding resistance to rice black stripe dwarf virus (RBSDV) [58]. To sum up, the candidate gene *Zm00001D006403* may influence photosynthesis and lead to the dysorganoplasia of tassel that follows infection by *Sporisorium reilianum*.

## 4. Materials and Methods

### 4.1. Plant and Sporisorium reilianum Materials

In the U.S., the maize inbred line Mo17 belongs to the Lancaster inbred group and is highly resistant to head smut, exhibiting a disease incidence of only 0–2% when infected by *Sporisorium reilianum*. Three maize inbred lines, showing different tassel symptoms in maize infected with *Sporisorium reilianum*, were used as plant materials: Huangzao4, Jing7, and Chang7-2 (Table 2). These three inbred lines all belong to the Sipingtou group. The inbred line Huangzao4 is widely used in China. Huangzao4 was bred and selected from natural mixed lines of Sipingtou. The inbred line Jing7 was bred and selected from the selfing of Jingzao7 (Huangzao4×luoxi3). The inbred line Chang7-2 was bred and selected from a single-cross hybrid Changdan7 (Huangzao4×Wei95). Huangzao4 has a head smut incidence rate of 92.31%, exhibits severe carbonization of tassels, and shows black filament with many spores (Figure 1b,c). Jing7 has a head smut incidence rate of 89.58%, with tassels expanding without spores (Figure 1e,f). Chang7-2 has a head smut incidence rate of 73.81%, and the base of its tassel is afflicted, whereas the upper part develops normally (Figure 1h,i).

Two F_2_ populations (Huangzao4×Jing7 and Jing7×Chang7-2) were constructed on May 2017 at the Xiangyang experimental base, Northeast Agricultural University, Harbin (45°45′36″ N, 126°55′48″ E). The two F2 groups were generated with 1389 pedigrees of Huangzao4×Jing7 and 592 pedigrees of the F_2_ populations of Jing7×Chang7-2.

The *Sporisorium reilianum* was collected from diseased plants in the autumn of 2016 at the Xiangyang experimental base of Northeast Agricultural University in Harbin. The teleutospore of *Sporisorium reilianum* was sieved through a 40-mesh screen. The teleutospore of *Sporisorium reilianum* was air-dried and stored at 20 °C in a dark room before it was used [9].

### 4.2. Analysis of Cell Morphological Changes in Tassels Infected with Sporisorium reilianum

The maize inbred lines Huangzao4, Jing7, Chang 7-2, and the resistance line Mo17 were artificially inoculated with *Sporisorium reilianum*. The *Sporisorium reilianum* of inoculated plants was detected with SR3 markers (5′-GCAGCCTCAGCATTACTC-3′, 5′-ATACACCTGTGACGGCTG-3′) [59]. The growing tips of the plant or tassel of the four maize inbred lines at VE, V2, V4, V6, V8, V10, and V12 stages were collected to analyze cell morphological changes. Collected samples were chopped into blocks of approximately 1 mm^3^, completely immersed in 2.5% glutaraldehyde for fixation, washed three times with 0.1 M PBS, fixed with 1% osmium acid for 2 h, dehydrated by an acetone gradient, and embedded by infiltration with an epoxy resin encapsulant. Samples were cut into ultrathin sections of 60–80 nm using an ultrathin sectioning machine, and double stained with uranyl acetate catalase and lead citrate to observe internal mycelial and cellular morphological changes, using a Hitachi 7650 TEM electron microscope (Hitachi High-Tech Science Co, Tokyo, Japan) [60].

### 4.3. Artificial Inoculation and Phenotypic Investigation

The soil was sieved through a 2 × 2 mm sieve and baked in an oven at 105 °C for 4–6 h. Water was then added, producing wet soil with an absolute moisture content of 20%. Full, uniformly sized seeds were disinfected with 1% sodium hypochlorite for 20 min and rinsed in distilled water five times for 4 h in a 37 °C water bath. The bottom of the paper tube was filled with 5 cm of wet soil, the seed embryo was placed face down on the soil, covered with about 2 g of 1% mycorrhizal soil of *Sporisorium reilianum*, and covered with about 2 cm of wet soil with an absolute moisture content of 20%. The seedlings were incubated in an artificial climate chamber at 20 °C with 8 h of light during the day, at 15 °C for 16 h at night, and then refined after 18 d. The seedlings were moved to the field at the V1 stage on 20 May 2017 [61]. The planting row spacing was 5 m, the distance between individual plants was 20 cm, and phenotypic investigation of each plant was conducted at the R3 stage.

The phenotype of the tassel symptoms for each plant (including parents and pedigrees) of the F_2_ populations were investigated as follows: Class A had typical symptoms of Huangzao4 with severe carbonization of the tassels and showed black filaments with many spores, Class B had typical symptoms of Jing7 accompanied by expanded tassels without spores, Class C had typical symptoms of Chang7-2, Class D had typical symptoms between Class A and B, and Class E had typical symptoms between Class B and C (Figure 1, Table 1 and Table 2).

### 4.4. Construction of a Mixed Gene Pool with Different Symptoms from the F_2_ Population

The phenotype of tassels of maize infected with *Sporisorium reilianum* from each plant from both F_2_ populations was investigated. Each line in the F_2_ population of Huangzao4×Jing7 was classified into three types: group A, B, or D. The mixed gene pool AB-A was built with 15 lines of the extreme phenotypes of A from the F_2_ populations of Huangzao4×Jing7. The gene pool AB-B was built with 15 lines from the extreme phenotypes of B from the F_2_ populations of Huangzao4×Jing7. For the F_2_ population of Jing7×Chang7-2, the lines were classified into three types: groups B, C, or E. The mixed gene pool BC-B was built with 15 lines of extreme phenotypes of A from the F_2_ populations of Jing7×Chang7-2. The gene pool BC-C was built with 15 lines from extreme phenotypes of B from the F_2_ populations of Jing7×Chang7-2.

### 4.5. DNA Extraction and Screening of SSR Markers

The DNA from each parent and the four mixed gene pools were extracted. DNA pools with pedigrees of four mixed gene pools were established. A total of 869 pairs of SSR markers covering the whole maize genome were selected from MaizeGDB (http://www.maizegdb.org, accessed on 28 October 2017). The polymorphisms of markers were performed between Huangzao4 and Jing7, and between Jing7 and Chang7-2. The polymorphism markers of two parents were used to detect the mixed gene pools of AB or BC. Twenty pairs of SSR markers were synthesized around the selected primers for polymorphism detection. Using the above markers, the phenotype of 270 lines from F_2_ populations of Huangzao4×Jing7 and 202 lines from F_2_ populations of Jing7×Chang7-2 were identified. This data was recorded for QTL analysis.

### 4.6. Data Processing and Statistical Analysis

The complete interval mapping method, using IciMapping 4.0, was used for QTL analysis, with a step size of 1 cM and 1000 regressions. The QTLs were mapped with a significance level of 0.05. Missing data was deleted.

In the F_2_ population of Huangzao4×Jing7, the types of electrophoretic bands of Huangzao4, Jing7, and the hybrid were marked as “2,” “0,” and “1,” respectively. In the F_2_ population of Jing7×Chang7-2, the types of electrophoretic bands of Jing7, Chang7-2, and the hybrid were marked as “2,” “0,” and “1,” respectively. Missing or fuzzy unrecognizable band-type marks were all marked as “−1”. LOD = 2.5 was used as a threshold to judge the QTLs. The QTL site was confirmed when a peak showed a LOD value that exceeded 2.5. The genetic effect and phenotypic variation of these QTLs were then calculated.

### 4.7. Candidate Genes Prediction and Bioinformatics Analysis

Candidate genes were predicted with differentially expressed protein analysis between Huangzao4 and Jing7, and Jing7 and Chang7-2 of label-free quantification proteome sequencing. Two candidate genes were analyzed using relevant databases and biological software, according to the following steps: (1) Intercept the nucleotide sequence before and after the reference sequence in NCBI, using the online prediction gene software Fgenesh (http://linux1.softberry.com/berry.phtml?topic=fgenesh&group=Programs&subgroup=gfind, accessed on 12 May 2022); (2) Select “FGENESH” in the “Gene Finding in Eukaryota” category on the main page of the software; (3) Use Monopot plants as a reference to predict the nucleotide sequence to obtain a gene with complete structure. Protparam (http://web.expasy.org/protparam/, accessed on 12 May 2022) was used to analyze the basic physical and chemical properties of the protein. SOPMA (http://npsa-pbil.ibcp.fr/cgi-bin/npsa_automat.pl?page=/NPSA/npsa_sopma.html, accessed on 12 May 2022) was used to analyze the secondary structure. The protein conservative domain, three-level structure diagram, homologous modeling, transmembrane structure, and signal peptide were analyzed with the Utilize InterPro online website (https://www.ebi.ac.uk/interpro/, accessed on 12 May 2022), Phyre (http://www.sbg.bio.ic.ac.uk/phyre/, accessed on 12 May 2022), DeepTMHMM Server v. 2.0 (https://dtu.biolib.com/DeepTMHMM, accessed on 12 May 2022), SignalP 5.0 (http://www.cbs.dtu.dk/services/SignalP/, accessed on 12 May 2022), and ClusterW (http://www.ebi.ac.uk/clustalw/, accessed on 12 May 2022). The homologous gene was found and downloaded to the NCBI database. The phylogenetics were analyzed with MEGAX software and the online website iTOL (https://itol.embl.de/, accessed on 12 May 2022).

## 5. Conclusions

The symptoms afflicting the tassels of maize infected with *Sporisorium reilianum* in inbred Sipingtou lines were significantly different and hereditarily stable. In this study, cytological analysis demonstrated that the cellular organization of three typical inbred lines (Huangzao4, Jing7, and Chang7-2) showed significant discrepancies at the VT stage. The QTLs, which control the symptoms of maize tassels infected with *Sporisorium reilianum*, were identified in two F_2_ populations using the BSA method with 869 SSR markers covering the whole maize genome. The results demonstrated that the QTL controlling the tassel symptoms in the Huangzao4 and Jing7 lines was located on bin 1.06, from markers umc1590 to bnlg1598, and explained 21.12% of the phenotypic variation with an additive effect of 0.6524. The QTL controlling the tassel symptoms in Jing7 and chang7-2 was located on bin 2.07 between markers umc1042 and bnlg1335, and explained 11.26% of the phenotypic variation with an additive effect of 0.4355. The genes *ZmABP2* and *Zm00001D006403* could be candidate genes for tassel symptoms of maize infected with *Sporisorium reilianum*.

## Figures and Tables

**Table 1:** Numbers of different tassel symptoms of maize infected with *Sporisorium reilianum* in two F_2_ populations.

Phenotype	Huangzao4×Jing7 F_2_ (Numbers)	Jing7×Chang7-2 F_2_ (Numbers)
A	265	-
B	234	146
C	-	183
D	890	263

**Table 2:** Information about three inbred lines with different symptoms affecting the tassels of maize infected with *Sporisorium reilianum*.

Inbred Lines	Types of Tassel Symptoms	Blood and Pedigree	Average Incidence	Tassel Symptoms
Huangzao4	A	Sipingtou group, widely used in China	92.31%	Exhibits severe carbonization of tassels and shows black filament with many spores
Jing7	B	Sipingtou group, selfing of Jingzao7 (Huangzao4×luoxi3)	89.58%	Tassels expand without spores
Chang7-2	C	Sipingtou group, single-cross hybrid Changdan7 (Huangzao4×Wei95)	73.81%	Base of its tassel is afflicted, whereas the upper part develops normally

## Data Availability

Not applicable.

## Supplementary Materials

The supporting information can be downloaded at: https://www.mdpi.com/article/10.3390/ijms232214416/s1.

## References

[1] Wang Y., Bao J., Wei X., Wu S., Fang C., Li Z., Qi Y., Gao Y., Dong Z., Wan X. (2022). Genetic structure and molecular mechanisms underlying the formation of tassel, Anther, and pollen in the male inflorescence of maize (*Zea Mays* L.). Cells.

[2] Alana P., Schirawski J. (2015). Host specificity in *Sporisorium reilianum* is determined by distinct mechanisms in maize and sorghum. Mol. Plant Pathol..

[3] Li Y., Wu X., Jaqueth J., Zhang D., Cui D., Li C., Hu G., Dong H., Song Y., Shi Y. (2015). The identification of two head smut resistance-related QTL in maize by the joint approach of linkage mapping and association analysis. PLoS ONE.

[4] Berke T.G., Rocheford T.R. (1999). Quantitative trait loci for tassel traits in maize. Crop Sci..

[5] Xu G., Wang X., Huang C., Xu D., Li D., Tian J., Chen Q., Wang C., Liang Y., Wu Y. (2017). Complex genetic architecture underlies maize tassel domestication. New Phytol..

[6] Li X., Wang Z., Gao S., Shi H., Zhang S., George M.L.C., Li M., Xie C. (2008). Analysis of QTL for resistance to head smut (*Sporisorium Reiliana*) in maize. Field Crop Res..

[7] Chen Y., Qing Q., Tan G., Zhao J., Zhang M. (2008). Identification and fine-mapping of a major QTL conferring resistance against head smut in maize. Theor. Appl. Genet..

[8] Weng J., Liu X., Wang Z., Wang J., Zhang L., Hao Z., Xie C., Li M., Zhang D., Li B. (2012). Molecular mapping of the major resistance quantitative trait locus qhs2.09 with simple sequence repeat and single nucleotide polymorphism markers in maize. Phytopathology.

[9] Zuo W., Zhao Q., Zhang N., Ye J., Tan G., Li B., Ye X., Zhang B., Liu H., Fengler K.A. (2015). A maize wall-associated kinase confers quantitative resistance to head smut. Nat. Genet..

[10] Di H., Yu T., Deng Y., Dong X., Li R., Zhou Y., Wang Z. (2017). Complementary DNA (cDNA) cloning and functional verification of resistance to head smut disease (*Sphacelotheca Reiliana*) of an NBS–LRR gene *ZmNL* in maize (*Zea Mays*). Euphytica.

[11] Lacaze A., Joly D.L. (2020). Structural specificity in plant-filamentous pathogen interactions. Mol. Plant Pathol..

[12] Shen Y., Liu N., Li C., Wang X., Xu X., Chen W., Xing G., Zheng W. (2017). The early response during the interaction of fungal phytopathogen and host plant. Open Biol..

[13] Rodenburg S., Seidl M.F., Judelson H.S., Vu A.L., Govers F., de Ridder D. (2019). Metabolic model of the phytophthora infestans-tomato interaction reveals metabolic switches during host colonization. mBio.

[14] Ghareeb H., Zhao Y., Schirawski J. (2018). *Sporisorium reilianum* possesses a pool of effector proteins that modulate virulence on maize. Mol. Plant Pathol..

[15] Li L., Zhu X., Zhang Y., Cai Y., Wang J., Liu M., Wang J., Bao J., Lin F. (2022). Research on the molecular interaction mechanism between plants and pathogenic fungi. Int. J. Mol. Sci..

[16] Doehlemann G., Ökmen B., Zhu W., Sharon A. (2017). Plant pathogenic fungi. Microbiol. Spectr..

[17] Zhang S., Li C., Si J., Han Z., Chen D. (2022). Action mechanisms of effectors in plant-pathogen interaction. Int. J. Mol. Sci..

[18] Pechanova O., Pechan T. (2015). Maize-pathogen interactions: An ongoing combat from a proteomics perspective. Int. J. Mol. Sci..

[19] Ghareeb H., Becker A., Iven T., Feussner I., Schirawski J. (2011). *Sporisorium reilianum* infection changes inflorescence and branching architectures of maize. Plant Physiol..

[20] Wang M., Yan J., Zhao J., Song W., Zhang X., Xiao Y., Zheng Y. (2012). Genome-wide association study (GWAS) of resistance to head smut in maize. Plant Sci..

[21] Khan M., Armin D. (2022). Performing infection assays of *Sporisorium reilianum F. Sp. Zeae* in maize. Methods Mol. Biol..

[22] Zhang S., Gardiner J., Xiao Y., Zhao J., Wang F., Zheng Y. (2013). Floral transition in maize infected with *Sporisorium reilianum* disrupts compatibility with this biotrophic fungal pathogen. Planta.

[23] Wu X., Li Y., Shi Y., Song Y., Zhang D., Li C., Buckler E.S., Li Y., Zhang Z., Wang T. (2016). Joint-linkage mapping and GWAS reveal extensive genetic loci that regulate male inflorescence size in maize. Plant Biotechnol. J..

[24] Upadyayula N., da Silva H.S., Bohn M.O., Rocheford T.R. (2006). Genetic and QTL analysis of maize tassel and ear inflorescence architecture. Theor. Appl. Genet..

[25] Ma B., Li Y., Duan S. (1983). Study on the resistence to head smut of corn varieties and its inheritance. Chin. Agric. Sci..

[26] Qiu C., Lv W., Lv D., Bai Y., Wei Q. (2014). Symptoms of four potato varieties infected with *Potato spindle tuber viroid* (Pstvd). Plant Prot..

[27] Fu S., Zhan Y., Zhi H., Gai J., Yu D. (2006). Mapping of SMV resistance gene *Rsc-7* by SSR markers in soybean. Genetica.

[28] Wu X., Pang Z., Tian L., Hu J. (1982). Physiological specialization of *Sphacelotheca reiliana (Kuhn) Clint.* to sorghum. Acta Phytopathol. Sin..

[29] Maya H., Mercado-Flores Y., Téllez-Jurado A., Pérez-Camarillo J., Mejía O., Anducho-Reyes M. (2020). Molecular variation of the phytopathogenic fungus *Sporisorium reilianum* in Valle del mezquital, Hidalgo. Front. Ecol. Evol..

[30] Shen X., Zhou M., Lu W., Ohm H. (2003). Detection of *Fusarium head blight* resistance QTL in a wheat population using bulked segregant analysis. Theor. Appl. Genet..

[31] Michelmore R.W., Paran I., Kesseli R.V. (1991). Dentification of markers linked to disease-resistance genes by bulked segregant analysis: A rapid method to detect markers in specific genomic regions by using segregating populations. Proc. Natl. Acad. Sci. USA.

[32] Subudhi P.K., Borkakati R.P., Virmani S.S., Huang N. (1997). Molecular mapping of a thermosensitive genetic male sterility gene in rice using bulked segregant analysis. Genome.

[33] Chen Q., Song J., Wen D., Xu L., Jiang Y., Zhang J., Xiang X., Yu G. (2017). Identification, mapping, and molecular marker development for *Rgsr8.1*: A new quantitative trait locus conferring resistance to *Gibberella* stalk rot in maize (*Zea Mays* L.). Front. Plant Sci..

[34] Bernier J., Kumar A., Ramaiah V., Spaner D., Atlin G. (2007). A large-effect QTL for grain yield under reproductive-stage drought stress in upland rice. Crop Sci..

[35] Salunkhe A.S., Poornima R., Prince K.S., Kanagaraj P., Sheeba J.A., Amudha K., Suji K.K., Senthil A., Babu R.C. (2011). Fine mapping QTL for drought resistance traits in rice (*Oryza sativa* L.) using bulk segregant analysis. Mol. Biotechnol..

[36] Altinkut A., Gozukirmizi N. (2003). Search for microsatellite markers associated with water-stress tolerance in wheat through bulked segregant analysis. Mol. Biotechnol..

[37] Lu H., Lin T., Klein J., Wang S., Qi J., Zhou Q., Sun J., Zhang Z., Weng Y., Huang S. (2014). QTL-seq identifies an early flowering QTL located near flowering locus T in cucumber. Theor. Appl. Genet..

[38] Chen W., Yao J., Chu L., Yuan Z., Li Y., Zhang Y. (2015). Genetic mapping of the nulliplex-branch gene (*gb_nb1*) in cotton using next-generation sequencing. Theor. Appl. Genet..

[39] Trick M., Adamski N.M., Mugford S.G., Jiang C.C., Febrer M., Uauy C. (2012). Combining SNP discovery from next-generation sequencing data with bulked segregant analysis (BSA) to fine-map genes in polyploid wheat. BMC Plant Biol..

[40] Ramirez-Gonzalez R.H., Segovia V., Bird N., Fenwick P., Holdgate S., Berry S., Jack P., Caccamo M., Uauy C. (2015). RNA-Seq bulked segregant analysis enables the identification of high-resolution genetic markers for breeding in hexaploid wheat. Plant Biotechnol. J..

[41] Veldboom L.R., Lee M., Woodman W.L. (1994). Molecular marker-facilitated studies in an elite maize population: I. Linkage analysis and determination of QTL for morphological traits. Theor. Appl. Genet..

[42] Ji H., Li X., Xie C., Hao Z., Lü X., Shi L., Zhang S. (2007). Comparative QTL mapping of resistance to *Sporisorium reiliana* in maize based on meta-analysis of QTL locations. J. Plant Res..

[43] Lu X., Brewbaker J.L. (1999). Molecular mapping of qtls conferring resistance to *Sphacelotheca reiliana (Kühn) Clint*. Maize Genet. Coop. Newsl..

[44] Lübberstedt T., Xia X., Tan G., Liu X., Melchinger A.E. (1999). QTL mapping of resistance to *Sporisorium reiliana* in maize. Theor. Appl. Genet..

[45] Shi H., Jiang Y., Wang Z., Li X., Zhang S. (2005). QTL identification of resistance to head smut in maize (in Chinese, English abstract). Acta Agron Sin..

[46] Di H., Liu X., Wang Q., Weng J., Zhang L., Li X., Wang Z. (2015). Development of SNP-based dCAPS markers linked to major head smut resistance quantitative trait locus qhs2.09 in maize. Euphytica.

[47] Wu Y., Vicente F.S., Huang K., Dhliwayo T., Costich D.E., Semagn K., Sudha N., Olsen M., Prasanna B.M., Zhang X. (2016). Molecular characterization of CIMMYT maize inbred lines with genotyping-by-sequencing SNPs. Theor. Appl. Genet..

[48] Zhang N., Zhang B., Zuo W., Xing Y., Konlasuk S., Tan G., Zhang Q., Ye J., Xu M. (2017). Cytological and molecular characterization of *ZmWAK*-mediated head-smut resistance in maize. Mol. Plant Microbe Interact..

[49] Gao S. (2005). Inheritance and quantitative trait loci mapping of resistance to Head Smut caused by *Sphacelotheca reiliana*(Kühn) in Maize. Ph.D. Thesis.

[50] Huang J., Sun W., Ren J., Yang R., Fan J., Li Y., Wang X., Joseph S., Deng W., Zhai L. (2020). Genome-wide identification and characterization of actin-depolymerizing factor (ADF) family genes and expression analysis of responses to various stresses in *Zea Mays* L.. Int. J. Mol. Sci..

[51] Burgos-Rivera B., Ruzicka D.R., Deal R.B., McKinney E.C., King-Reid L., Meagher R.B. (2008). Actin depolymerizing factor9 controls development and gene expression in Arabidopsis. Plant Mol. Biol..

[52] Lopez I., Anthony R.G., Maciver S.K., Jiang C.J., Khan S., Weeds A.G., Hussey P.J. (1996). Pollen specific expression of maize genes encoding actin depolymerizing factor-like proteins. Proc. Natl. Acad. Sci. USA.

[53] Kurepa J., Smalle J.A. (2019). Oxidative stress-induced formation of covalently linked ribulose-1,5-bisphosphate carboxylase/oxygenase large subunit dimer in tobacco plants. BMC Res. Notes.

[54] Doron L., Segal N., Gibori H., Shapira M. (2014). The BSD_2_ ortholog in Chlamydomonas reinhardtii is a polysome-associated chaperone that co-migrates on sucrose gradients with the rbcL transcript encoding the Rubisco large subunit. Plant J..

[55] Schulz L., Guo Z., Zarzycki J., Steinchen W., Schuller J.M., Heimerl T., Prinz S., Mueller-Cajar O., Erb T.J., Hochberg G. (2022). Evolution of increased complexity and specificity at the dawn of form I Rubiscos. Science.

[56] Grabsztunowicz M., Górski Z., Luciński R., Jackowski G. (2015). A reversible decrease in ribulose 1,5-bisphosphate carboxylase/oxygenase carboxylation activity caused by the aggregation of the enzyme’s large subunit is triggered in response to the exposure of moderate irradiance-grown plants to low irradiance. Physiol. Plant..

[57] Qu Y., Sakoda K., Fukayama H., Kondo E., Suzuki Y., Makino A., Terashima I., Yamori W. (2021). Overexpression of both rubisco and rubisco activase rescues rice photosynthesis and biomass under heat stress. Plant Cell Environ..

[58] Feng Z., Yuan M., Zou J., Wu L., Wei L., Chen T., Zhou N., Xue W., Zhang Y., Chen Z. (2021). Development of marker-free rice with stable and high resistance to rice black-streaked dwarf virus disease through RNA interference. Plant Biotechnol. J..

[59] Xu M.L., Melchinger A.E., Lübberstedt T. (1999). Species-specific detection of the maize pathogens *Sporisorium reiliana* and *Ustilago maydis* by dot blot hybridization and PCR-based assays. Plant Dis..

[60] Qi F., Zhang L., Dong X., Di H., Zhang J., Yao M., Dong L., Zeng X., Liu X., Wang Z. (2019). Analysis of cytology and expression of resistance genes in maize infected with *Sporisorium Reilianum*. Plant Dis..

[61] Wang Z., Sun P., Li N., Di H., Wang Y., Liu X., Zhang L., Yu T. (2015). Indoors infecting factors optimization of maize head smut under seedling stage. J. Northeast Agric. Univ..

