# Supplementary material for: Analysis of QTLs and Candidate Genes for Tassel Symptoms in Maize Infected with Sporisorium reilianum
Source: Int J Mol Sci. 2022 Nov 20;23(22):14416. doi: 10.3390/ijms232214416 (PMC9692487; doi:10.3390/ijms232214416)
Supplement: Supplementary file 1 [file ijms-23-14416-s001.zip › Supplementary Figures S1-S3.pdf]

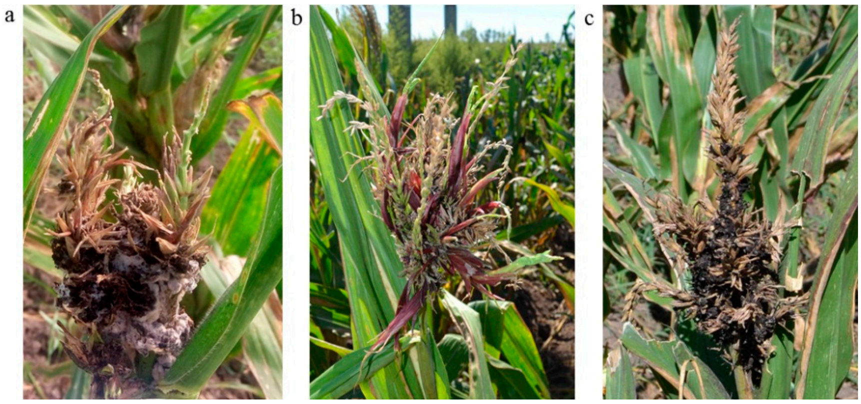

Supplementary Figure S1: The intermediate symptoms of tassel of typical plants infected with *Sporisorium reilianum* in F_2 Populations. a. The intermediate symptoms of Huangzao4 × Jing7; b. The intermediate symptoms of Huangzao4 × Jing7; c. The intermediate symptoms of Jing7×Chang7-2

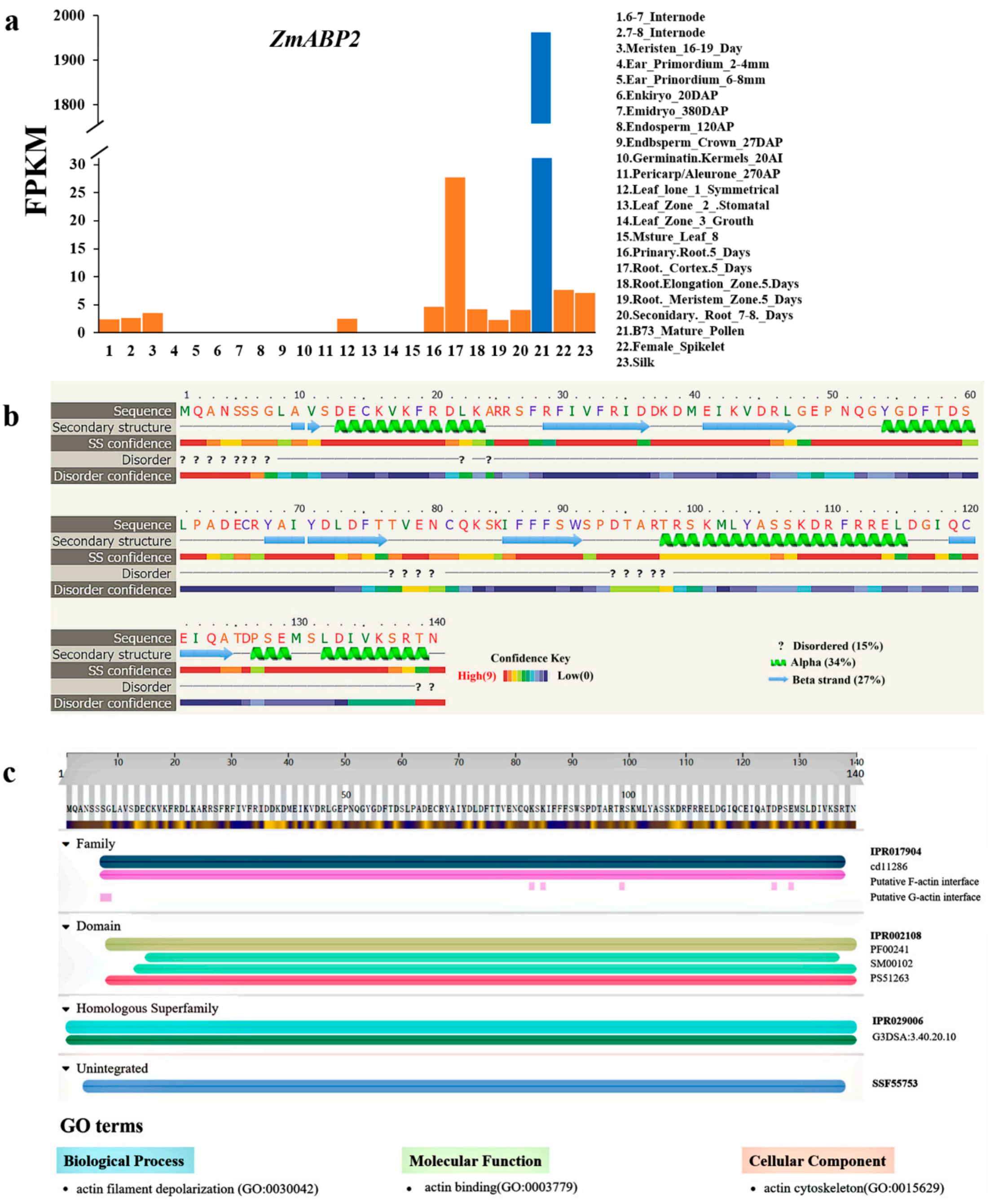

Supplementary Figure S2: Gene expression and protein structure of ZmABP2. a. Prediction of spatial and temporal expression of *ZmABP2* gene. b.Secondary structure prediction of ZmABP2 protein. c. The conserved domains of ZmABP2 protein.

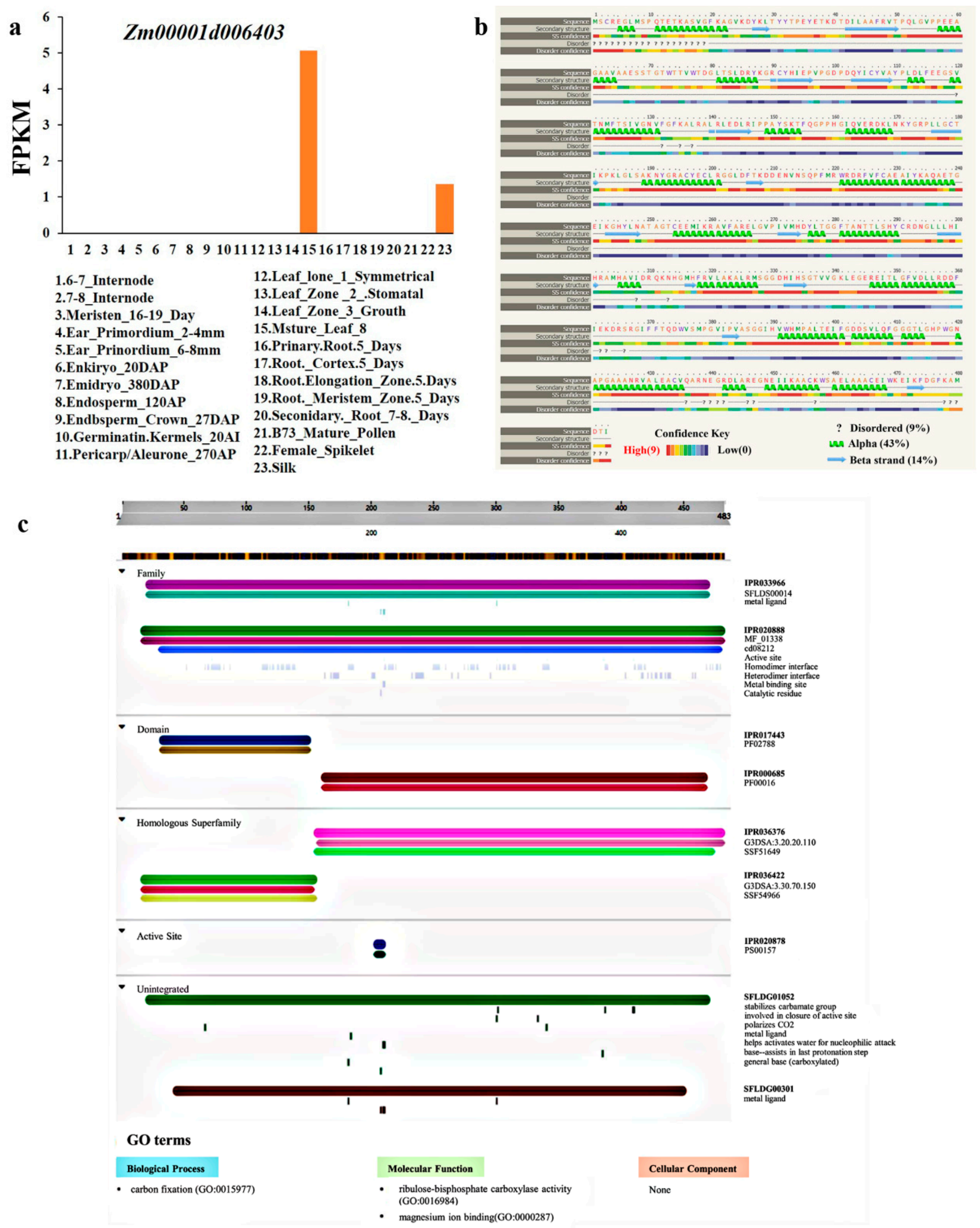

Supplementary Figure S3: Gene expression and protein structure of Zm00001d006403. a. Prediction of spatial and temporal expression of *Zm00001d006403* gene. b. Secondary structure prediction of Zm00001d006403 protein. c. The conserved domains of Zm00001d006403 protein.
