# Supplementary material for: Analysis of QTLs and Candidate Genes for Tassel Symptoms in Maize Infected with Sporisorium reilianum
Source: Int J Mol Sci. 2022 Nov 20;23(22):14416. doi: 10.3390/ijms232214416 (PMC9692487; doi:10.3390/ijms232214416)
Supplement: Supplementary file 1 [file ijms-23-14416-s001.zip › Supplementary Table S1.pdf]

Supplementary Table S1. Detailed information of polymorphic primers

Primer	Primer sequences (5'-3')	Chromosome location	Map position	Physical location	
umc1395	TGAATGAGTGGCATTCAAAATCTG<br>CAGATTGCATGTGTGAGTGTGTGT	bin1.05	471.7	164572134	164574375
umc1601	TTTTTACAGCAGGAAGAAGGCTTTA<br>TAATGGCATGGCATGTAGGATCT	bin1.05	473.8	166785190	166789349
umc1323	TTGCACCCCGTTATTATCCTACAG<br>CCAGACTAGAGTGCCATGATCCTT	bin1.05	484.72	170491369	170495919
umc1754	ATAGGGATCGACCCGTTCGT<br>AATATCTCCGATCCACCAACAAAA	bin1.06	506.83	180248365	180709406
umc1590	CAGAGTCTGATAGTCCGAACCCAG<br>GTAAAGCTCACAGCTTCCGACAG	bin1.06	517	182873055	182876103
umc1811	AGATAGCCGCCGAGACCAAG<br>ACTCACTCGACGGACTTCTCGAC	bin1.06	526.4	184732930	184733551
bnlg2057	CAGCAGAACCTGTGGACAGA<br>TGCATACTTGAGGATCGGAG	bin1.06	527.6	184813331	184814965
bnlg1598	GGCAAGATTCGGACCAGG<br>CGGTTAGGAGCAGTACGTCA	bin1.06	532.8	187975047	188132153
bnlg1914	ATGCAACATTTCGTGATCCA<br>GATTTTTCTAGCACTCGCGC	bin2.05	370	151448752	152415060
umc2019	GACATGGACTGCCTTCAAATGAT<br>ATAGCTTTTCTCAGTAAGCGCCAG	bin2.06	406.14	186410808	187894598
umc2205	ATGGTGAGCGAGTGAAAGAGAGAT<br>CATGATCATTTGGCGATGGTAAT	bin2.07	435.82	192589913	195782417
bnlg1335	GAAGGTTGCTCTTCCACTGG	bin2.07		198482067	199166751

	TGGTTTGTGCAAGTGTCACC		456.09		
umc1042	AAGGCACTGCTACTCCTATGGCTA<br>CTGACCTTTGAATTCTGTGCTCCT	bin2.07	466.65	202213552	202371807
